# Manual of Physical Diagnosis

**Published:** 1902-04

**Authors:** 


MANUAL OF PHYSICAL DIAGNOSIS. For the use of Students and Physicians. By JAMES TYSON, M. D., Professor of Medicine in the University of Pennsylvania and Physician to the University Hospital; Physician to the Philadelphia Hospital; Fellow of the College of Physicians of Philadelphia; Member of the Association of American Physicians, etc. Fourth edition, revised and enlarged. Duodecimo. With colored and other illustrations. Philadelphia: P. Blakiston's Son & Co. 1901. [Price, cloth, $1.50 net.]

Tyson's physical diagnosis is well known to the profession as a careful, accurate guide in the study of the diseases of the different organs of the body. Although not so complete as the works of Musser, Hare, Simon or von Jaksch, it, nevertheless, fills a want long ago felt by the practising physician, and will continue to gain favor with each succeeding edition. The present edition has been carefully revised and somewhat enlarged to meet the demands of the hour. It is similar in appearance to former editions. W.C.K.

